# Usefulness of the Brief Scale for Psychiatric Problems in Orthopaedic Patients (BS-POP) for Predicting Poor Outcomes in Patients Undergoing Lumbar Decompression Surgery

**DOI:** 10.1155/2021/2589865

**Published:** 2021-12-21

**Authors:** Kazuyuki Watanabe, Koji Otani, Takuya Nikaido, Kinshi Kato, Hiroshi Kobayashi, Junichi Handa, Shoji Yabuki, Shin-Ichi Kikuchi, Shin-Ichi Konno

**Affiliations:** Department of Orthopaedic Surgery, Fukushima Medical University School of Medicine, Fukushima, Japan

## Abstract

**Background:**

The Brief Scale for Psychiatric Problems in Orthopaedic Patients (BS-POP) is an original questionnaire that evaluates psychosocial problems in orthopaedic patients. The purpose of this study was to clarify the relationship between BS-POP scores and surgical outcomes in patients with lumbar spinal stenosis (LSS).

**Methods:**

From our database, a total of 157 patients with LSS who had undergone decompression surgery and completed a 1-year follow-up were retrospectively observed. The primary outcome was the numerical rating scale (NRS) score for satisfaction with surgery (from 0: not satisfied to 10: completely satisfied). Patients with an NRS score ≥8 were classified into the satisfied group. The secondary outcomes were NRS scores for low back pain, leg pain, and leg numbness and scores on the Roland–Morris Disability Questionnaire (RDQ). BS-POP was used to detect psychiatric problems before surgery. A BS-POP score ≥11 on the physician version or a combination of 10 on the physician version and ≥15 on the patient version was considered to indicate the presence of psychiatric problems. The patients were classified into two groups and compared based on preoperative BS-POP scores at the 1-year follow-up.

**Results:**

Preoperatively, 22 and 135 patients showed high and low BS-POP scores, respectively. No significant differences in preoperative symptoms were found between the two groups. At 1 year after surgery, patients with high BS-POP scores showed significantly lower satisfaction with surgery, higher NRS scores for low back pain, leg pain, and leg numbness, and lower RDQ deviation scores than did the low BS-POP group (*p* < 0.05). The results of the multivariable analysis indicated that preoperative high BS-POP scores were independently associated with low satisfaction with surgery (odds ratio: 5.2, 95% confidence interval: 1.9–15.1).

**Conclusion:**

High preoperative BS-POP scores were associated with poor outcomes for decompression surgery in patients with LSS at 1 year after surgery. These results suggest that BS-POP is a useful tool for predicting surgical outcomes in patients with LSS.

## 1. Introduction

Psychiatric problems have been reported to be related to poor surgical outcomes in patients with lumbar spinal disorders [1]. Various depression scales and questionnaires, including the Minnesota Multiphasic Personality Inventory (MMPI) [2–5], have been used in previous studies to analyze the relationship between depression and lumbar surgery outcomes [6–15]. Psychological distress has also been reported to be a predictive factor of a poor surgical outcome [13, 16]. In addition, fear-avoidance beliefs have been shown to be a baseline psychological factor that significantly predicts negative lumbar surgery outcomes, whereas psychological disturbances, including depression, do not significantly predict outcomes in the same study [17]. Therefore, the value of psychiatric factors for predicting poor surgical outcomes, as well as what kind of and how many problems should be evaluated as risk factors, remains controversial for patients with lumber spinal disorders.

Surgeons sometimes find it difficult to treat patients with personality problems and excessive concern about their symptoms. However, to our knowledge, no tool has been developed to evaluate such problems objectively. The MMPI is one of the most commonly used tools for analyzing psychiatric problems, including hysteria, hypochondria, and depression [18]. This type of multidimensional analysis regarding psychiatric status may be useful for predicting surgical outcomes. However, the MMPI takes from 40 minutes to 1 hour to complete and requires an experienced psychologist or psychiatrist to conduct the subsequent analysis; it is difficult for surgeons to analyze. Useful self-administered questionnaires are available for assessing depression, including the Zung Self-Rating Depression Scale [6–8, 14, 17] and Beck Depression Inventory [11, 15]. Although these tools provide substantial amounts of evidence for patients with lumbar spinal disorders, we considered that not only depression but also other psychiatric problems, such as personality problems, are essential for the evaluation of such patients preoperatively. For these reasons, the Brief Scale for Psychiatric Problems in Orthopaedic Patients (BS-POP) was developed as an original questionnaire to evaluate psychiatric problems in clinical settings [19–21]. The BS-POP has two components: one for the physician and another for the patient (Supplementary Materials). The physician version is composed of 8 questions, and physicians answer each question based on their assessment of the patient. Each question is assessed on a 3-point scale, with total scores ranging from 8 to 24 points, and higher scores indicating worse problems. The patient version of BS-POP is composed of 10 questions and is completed by patients to assess their mood problems. Each item is assessed on the same scale as the physician version, with total scores ranging from 10 to 30 points, and higher scores indicating more severe psychiatric problems. Validation and responsiveness studies for BS-POP have been conducted [20–22], and this questionnaire is now routinely used in our institutional hospital to evaluate patients undergoing spinal surgery preoperatively. The purpose of this study was to clarify the relationship between preoperative BS-POP scores and lumbar surgery outcomes and the usefulness of BS-POP.

## 2. Materials and Methods

From 2003, we developed a database for prospectively recording the outcomes of spine surgery in our institution; this study was a retrospective study using this database. From the database, we enrolled 229 patients with lumbar spinal stenosis (LSS) who had undergone lumbar decompression surgery from 2003 to 2011. Patients who had not completed preoperative evaluations and a 1-year follow-up were excluded, as were those who underwent fusion surgery or second operations and those with coexisting cervical or thoracic spinal disease, rheumatoid arthritis, and destructive spondyloarthropathy. Finally, 157 patients were analyzed in this study.

The preoperative examinations consisted of the BS-POP (≥11 points on the physician version or a combination of 10 points on the physician version and ≥15 points on the patient version were considered high scores [19, 22]), a numerical rating scale (NRS) for low back pain, leg pain, and leg numbness (scores range from 0 to 10, with higher scores indicating worse pain), and the Roland–Morris Disability Questionnaire (RDQ) [23]. For patients aged 20–79 years, the norm-based RDQ score was calculated using the RDQ mean (baseline) and standard deviation by gender and age of complainant with low back pain from a national survey in Japan [24]. The national norm was converted to 50 points and the standard deviation to 10. Deviation scores higher than 50 indicate a better quality of life than the national average, whereas scores lower than 50 indicate worse quality of life than the national average. For preoperative radiological assessments, the presence of spondylolisthesis (grade I or higher), degenerative scoliosis (cobb angle >10°), and lumbar kyphosis (lumbar lordosis <0°) were examined using X-rays of the lumbar spine in the standing position. For neurological assessments, the presence of cauda equina symptoms such as intermittent claudication with numbness of both legs, numbness of both soles at rest, and bowel/bladder dysfunction due to LSS were evaluated. The primary outcome was the NRS score for satisfaction with surgery at 1-year follow-up (from 0: not satisfied to 10: completely satisfied). Patients with an NRS score ≥8 and < 8 were classified into the satisfied and dissatisfied groups, respectively. The secondary outcomes were NRS scores for symptoms and scores on the RDQ. This study was approved by the Ethics Committee of Fukushima Medical University (reference no.: 4089). Informed consent was obtained in the form of opt-out on the website.

### 2.1. Statistical Analysis

The patients were divided into two groups according to preoperative BS-POP scores (high and low score groups) and compared. The chi-square and Wilcoxon tests were used for the univariable analysis. Logistic regression analysis was conducted to investigate the relationship between preoperative BS-POP scores and surgical outcomes, with the other preoperative assessments considered as confounding factors. Cauda equina symptoms were investigated as an indicator of neurological severity. Lumbar spinal deformities (spondylolisthesis, scoliosis, and kyphosis) were also investigated as candidates for confounding factors as they affect the outcome of decompression surgery. Statistical analysis was carried out using JMP software (ver. 15.0; SAS Institute, Cary, NC). Values of *p* < 0.05 were considered significant.

## 3. Results

Based on the preoperative BS-POP scores, 22 and 135 patients were classified into the high and low BS-POP groups, respectively (Table 1). According to preoperative X-rays of the lumbar spine, 82 patients were found to have degenerative spondylolisthesis, 14 with lumbar kyphosis, and 30 with lumbar scoliosis; in addition, cauda equina symptoms were found in 90 patients (Table 1). The high BS-POP group included significantly more female patients than did the low BS-POP group (*p* < 0.01). No significant differences in the proportion of patients with spondylolisthesis, lumbar kyphosis, or lumbar scoliosis were found between the two groups. Regarding neurological findings, no significant difference in the percentage of patients with cauda equina symptoms was found. Regarding preoperative symptoms, no significant differences in NRS scores for low back pain, leg pain, or leg numbness were observed between the two groups (Table 1). On the preoperative RDQ evaluation, the high BS-POP group had a significantly higher total score than the low BS-POP group, but no significant difference was seen in the norm-based RDQ score (Table 1). At 1 year after surgery, the high BS-POP group showed significantly lower NRS scores for satisfaction with surgery than did the low BS-POP group (Table 2). In addition, the rate of satisfaction was significantly lower in the high BS-POP group than in the low BS-POP group (Table 2). The high BS-POP group had higher NRS scores than the low BS-POP group for low back pain, leg pain, and leg numbness (Table 2). In the high BS-POP group, the mean norm-based RDQ score was 35.1 before surgery and improved to 44.1 after surgery; however, this score was still significantly lower than that in the low BS-POP group (mean score: 50) (Table 2). In the univariate analysis investigating the relationship between lower satisfaction and preoperative factors, the presence of preoperative high BS-POP scores and cauda equina symptoms was significantly associated with lower satisfaction with surgery (*p* < 0.05) (Table 3). Based on these results, cauda equina symptoms were included in the multivariate analysis as a confounding factor, in addition to age and gender. The results of the multivariate analysis indicated that preoperative high BS-POP scores were independently associated with low satisfaction with surgery (odds ratio: 5.2; 95% confidence interval: 1.9–15.1) after adjusting for age, gender, and neurological type (Table 4).

## 4. Discussion

In this study, preoperative BS-POP scores were associated with poor outcomes for lumbar decompression surgery. Although NRS scores for each symptom and RDQ scores were improved in both groups, patients in the high BS-POP group had significantly lower satisfaction and worse symptoms at 1 year after surgery than did those in the low BS-POP group. Regarding the clinical implications of these findings, preoperative BS-POP scores could help identify patients at risk of a poor outcome for lumbar decompression surgery.

The mechanisms by which psychiatric factors affect surgical outcomes are not fully understood. In lumbar spinal disorders, chronic low back pain is reported to be closely associated with depressive and anxiety states [25], and such psychiatric conditions are exacerbated by chronic low back pain [26]. The worse the psychiatric state, the more exacerbated the low back pain becomes, leading to a vicious cycle [27, 28]. Low back pain with psychiatric problems is difficult to treat by standard therapies, including surgery, which partially explains why psychiatric problems are one of the causes of “failed back” syndrome [29]. A possible explanation for dissatisfaction based on high BS-POP scores is that the BS-POP score is related to personality problems and excessive concern about symptoms [19, 21]. Thus, satisfaction may be reduced because of excessive dissatisfaction with residual symptoms after surgery.

Similar to the present study, psychiatric problems have been reported to be predictors of poor outcomes in decompression surgery for LSS [9–11, 15, 17]. Regarding depression, which is a typical psychiatric problem, Sinikallio et al. reported that preoperative depression and continuous postoperative depression were predictors of poor outcomes at 2 years after lumbar decompression surgery for LSS [11]. Tuomainen et al. performed a longer follow-up study and found that preoperative depression was associated with poor outcomes at 10-year follow-up assessments [15]. These findings suggest that to improve surgical outcomes, it is important to assess depression preoperatively.

The BS-POP was developed to enable surgeons to assess psychiatric problems more easily [19]. The physician and patient versions of BS-POP are composed of 8 and 10 questions, respectively, and only takes a few minutes to complete. Another advantage of BS-POP is that it consists of two components. Self-reported questionnaires are typically used to assess psychiatric problems. In addition to patient self-assessments, BS-POP includes objective assessments by physicians, which allows a more accurate evaluation of psychiatric problems [19, 21].

In the high BS-POP group, there were three possible score combinations (≥11 points on the physician version and ≥14 points on the patient version: 6 cases; ≥11 points on the physician version and ≥15 points on the patient version: 6 cases; or a combination of 10 points on the physician version and ≥15 points on the patient version: 10 cases). In this study, we did not find any differences in primary and secondary outcomes among the groups; however, due to the small number of cases in the high BS-POP group, further study is needed to determine if there is a difference in surgical outcomes because of the combination of BS-POP scores.

In this study, the high BS-POP group included significantly more female patients than did the low BS-POP group. The BS-POP includes some questions items on mood problems and depression. Since women are well known to have a higher risk for depressive symptoms than men [30], more female patients might have been in the high BS-POP group. In the preoperative evaluations, the high BS-POP group showed a significantly lower RDQ score than did the low BS-POP group. On the other hand, there was no statistically significant difference in the norm-based RDQ score. These results suggested no significant difference between the two groups in the evaluation of preoperative RDQ when adjusted for age and gender.

At 1 year after surgery, the high BS-POP group had more residual symptoms and lower satisfaction with surgery than did the low BS-POP group. Thus, surgeons should check for the presence of psychiatric problems in patients with a high BS-POP score when assessing the risk of a poor outcome. For such patients, one option is to avoid surgery. However, in cases with uncontrolled pain, severe paralysis, or bowel/bladder dysfunction, surgery may be necessary. In such cases, for whom surgical results may be worse than expected, additional informed consent may be needed from patients and their families.

The results of this study revealed that apart from psychiatric problems, cauda equina symptoms were also associated with lower satisfaction with surgery. This finding suggests that caudal symptoms are more likely to persist after decompression surgery. In addition, cauda equina symptoms such as bowel/bladder dysfunction and numbness of both soles are known to reduce the quality of life of patients with LSS [31]. Therefore, patients with cauda equina symptoms should be considered for surgery as soon as possible before becoming severely ill.

This study had several limitations. First, only cases that could be followed for 1 year were included, so the possibility of selection bias cannot be ruled out. In addition, another selection bias could have occurred, as high-risk patients with clear psychiatric problems may have been excluded before surgery. Second, the effects of postoperative treatment for residual symptoms, such as medication and rehabilitation, which may affect outcomes, were not assessed. Third, some patients showed a change in psychiatric status after surgery, and depression and psychological disturbances have been shown to improve postoperatively in patients with good outcomes [11, 17]. The BS-POP shows good responsiveness in terms of the treatment of patients with chronic low back pain [22] and LSS [32]. Therefore, additional research is needed to clarify further the relationship between changes in BS-POP scores after surgery and surgical outcomes. Fourth, there was no further evaluation of the patients with high BS-POP scores. Therefore, what actual diagnosis they received from a psychiatrist remains unclear. In future studies, patients with high BS-POP scores need to be examined by a psychiatrist. Fifth, the follow-up period was 1 year in this study, which might be relatively short to discuss operative outcomes. Further follow-up could reveal differences in operative outcomes. This study used the Japanese version of the BS-POP (the English version is given in Supplementary Materials). Although validation studies of the Japanese version of the BS-POP have been reported [20–22], for English-speaking countries, further investigations of the validity and reliability of the English version of the BS-POP are needed.

## 5. Conclusion

The results of this study revealed that preoperative high BS-POP scores were related to poor outcomes for LSS at 1 year after surgery. Therefore, BS-POP scores may help predict the risk of poor surgical outcomes for patients with LSS. When preoperative BS-POP scores are high, we recommend checking for the presence of psychiatric problems and carefully reconsidering the indication for surgery.

## Figures and Tables

**Table 1:** Preoperative demographic data, NRS symptom scores, and RDQ scores.

	Low BS-POP	High BS-POP	*P* value
*N*	135	22	
Male/female	86/49	8/14	0.02
Mean age (y)	67.3 ± 10.3	66.5 ± 9.9	0.79
Spondylolisthesis (+/−)	70/65	12/10	0.81
Degenerative scoliosis (+/−)	25/110	5/17	0.65
Lumbar kyphosis (+/−)	11/124	3/19	0.43
Cauda equina symptoms (+/−)	80/55	10/12	0.22
Symptoms			
Low back pain	4.2 ± 3.2	4.1 ± 3.0	0.81
Leg pain	4.7 ± 3.6	5.8 ± 3.1	0.28
Leg numbness	5.8 ± 3.2	5.8 ± 3.3	0.94
RDQ			
Total score	10.9 ± 5.5	13.9 ± 5.4	0.03
Norm-based score	38.5 ± 11.2	35.2 ± 9.9	0.17

Results are expressed as mean ± standard deviation for NRS and RDQ scores. RDQ, Roland–Morris Disability Questionnaire; BS-POP, Brief Scale for Psychiatric Problems in Orthopaedic Patients; NRS, numerical rating scale.

**Table 2:** Satisfaction, NRS symptom scores, and RDQ scores at the 1-year follow-up.

	Low BS-POP	High BS-POP	*P* value
*N*	135	22	
Satisfaction	8.3 ± 2.0	7.2 ± 2.7	0.02
Satisfied group (+/−)	100/35	10/12	0.01
Symptoms			
Low back pain	1.8 ± 2.3	4.2 ± 3.3	<0.01
Leg pain	1.5 ± 2.5	3.5 ± 3.4	0.01
Leg numbness	2.9 ± 3.0	4.3 ± 3.3	0.04
RDQ			
Total score	5.9 ± 5.4	9.1 ± 6.0	0.02
Norm-based score	49.4 ± 8.9	44.1 ± 10.5	0.04

Results are expressed as mean ± standard deviation for NRS and RDQ scores. RDQ, Roland–Morris Disability Questionnaire; BS-POP, Brief Scale for Psychiatric Problems in Orthopaedic Patients; NRS, numerical rating scale.

**Table 3:** Preoperative status and satisfaction at 1 year after surgery.

	Satisfied group	Dissatisfied group	*P* value
*N*	110	47	
Male/female	64/46	30/17	0.51
Mean age (*y*)	66.6 ± 10.8	68.7 ± 8.5	0.39
Spondylolisthesis (+/−)	55/55	27/20	0.39
Degenerative scoliosis (+/−)	21/89	9/38	0.99
Lumbar kyphosis (+/−)	10/100	4/43	0.91
Cauda equina symptoms (+/−)	57/53	33/14	0.03
High BS-POP score (+/−)	10/100	12/35	<0.01

BS-POP, Brief Scale for Psychiatric Problems in Orthopaedic Patients.

**Table 4:** Regression analysis of risk factors for low satisfaction with lumbar decompression surgery.

Variable	Odds ratio (95% CI)	*P* value
High BS-POP score	5.2 (1.9–15.1)	<0.01
Cauda equina symptoms	2.5 (1.2–5.7)	0.02
Age (≥70 y)	1.5 (0.7–3.2)	0.26
Female	0.6 (0.3–1.2)	0.15

BS-POP, Brief Scale for Psychiatric Problems in Orthopaedic Patients; CI, confidence interval.

## Data Availability

The data used to support the findings of this study are available from the corresponding author upon request.

## Supplementary Materials

Supplementary Materials These are the questionnaires for medical personnel (BS-POP for use by physicians) and patients (BS-POP for use by patients).
