# Supplementary material for: Usefulness of the Brief Scale for Psychiatric Problems in Orthopaedic Patients (BS-POP) for Predicting Poor Outcomes in Patients Undergoing Lumbar Decompression Surgery
Source: Pain Res Manag. 2021 Dec 21;2021:2589865. doi: 10.1155/2021/2589865 (PMC8714325; doi:10.1155/2021/2589865)
Supplement: Supplementary Materials — These are the questionnaires for medical personnel (BS-POP for use by physicians) and patients (BS-POP for use by patients). [file 2589865.f1.doc]

**Supplemental files**

**Questionnaire for medical personnel (BS-POP for use by physicians), English version**

| Questions | Responses and scores | | |
| --- | --- | --- | --- |
| 1. The patient’s pain appears uninterrupted | 1 That is not the case | 2 The pain is intermittent | 3 The patient appears to be in pain almost always |
| 2. The patient has a specific way of indicating the symptomatic area(s) | 1 That is not the case | 2 The patient rubs the symptomatic area(s) | 3 Without instruction, the patient removes their clothes and shows the symptomatic area(s) |
| 3. The patient appears to have pain over the whole symptomatic area | 1 That is not the case | 2 Sometimes | 3 Almost all the time |
| 4. When examination or treatment is recommended, the patient becomes badly tempered, easily angered, or argumentative | 1 That is not the case | 2 The patient shows slight resistance | 3 The patient shows significant resistance |
| 5. When having their senses assessed, the patient responds excessively to stimulation | 1 That is not the case | 2 The patient’s response is slightly excessive | 3 The patient’s response is quite excessive |
| 6. The patient repeatedly asks questions regarding their condition or surgery | 1 That is not the case | 2 Sometimes | 3 Almost all the time |
| 7. The patient changes their attitude depending on the medical staff member | 1 That is not the case | 2 Somewhat | 3 Significantly |
| 8. The patient wishes that their symptoms were gone, even slight symptoms | 1 That is not the case | 2 Somewhat | 3 Significantly |

**Notes:** Numbers next to the responses indicate scores. **Abbreviations:** BS-POP, Brief Scale for Psychiatric Problems in Orthopaedic Patients.

| **Questionnaire for patients (BS-POP for use by patients), English version** | | | |
| --- | --- | --- | --- |
| Questions | Responses and scores | | |
| 1. Do you ever feel like crying, or do you cry? | 1 No | 2 Sometimes | 3 Almost all the time |
| 2. Do you always feel miserable and unhappy? | 1 No | 2 Sometimes | 3 Almost all the time |
| 3. Do you always feel nervous and irritated? | 1 No | 2 Sometimes | 3 Almost all the time |
| 4. Do you feel annoyed and aggravated over small things? | 1 No | 2 Sometimes | 3 Almost all the time |
| 5. Do you have a normal appetite? | 3 No | 2 Sometimes | 1 Almost all the time |
| 6. Are you in your best mood in the morning? | 1 No | 2 Sometimes | 3 Almost all the time |
| 7. Do you get somewhat tired? | 1 No | 2 Sometimes | 3 Almost all the time |
| 8. Are you able to put your usual effort into your work? | 1 No | 2 Sometimes | 3 Almost all the time |
| 9. Do you feel satisfied with the sleep you are getting? | 3 No | 2 Sometimes | 1 Almost all the time |
| 10. Do you have trouble falling asleep for any reason other than pain? | 1 No | 2 Sometimes | 3 Almost all the time |

**Notes:** Numbers next to the responses indicate scores. **Abbreviations:** BS-POP, Brief Scale for Psychiatric Problems in Orthopaedic Patients.
